# ‘Oh God, I Have to Eat Something, But Where Can I Get Something Quickly?’—A Qualitative Interview Study on Barriers to Healthy Eating among University Students in Germany

**DOI:** 10.3390/nu11102440

**Published:** 2019-10-14

**Authors:** Jennifer Hilger-Kolb, Katharina Diehl

**Affiliations:** Mannheim Institute of Public Health, Social and Preventive Medicine, Medical Faculty Mannheim, Heidelberg University, 68167 Mannheim, Germany; Katharina.Diehl@medma.uni-heidelberg.de

**Keywords:** university students, barriers, eating behavior, qualitative research

## Abstract

Healthy eating can prevent individuals across all age groups from developing overweight/obesity and non-communicable diseases such as type 2 diabetes and cardiovascular disease. However, unhealthy eating habits (e.g., a high level of fast food consumption) have been found to be widespread among university students. Thus, it seems necessary to develop prevention strategies to improve students’ eating habits. However, to ensure that such strategies are successful, it is important that they fit the needs of the target population. By conducting qualitative interviews with students (*n* = 20), we aimed to get a deeper understanding of barriers to healthy eating. Students were asked about barriers to healthy eating and to suggest possible ideas that could improve their eating behavior in the future. Our findings revealed that students are especially affected by time-related barriers (e.g., a lack of time due to university commitment) and environmental barriers (e.g., a lack of cheap, tasty, and healthy meal options at the university canteen). Time-related barriers were also related to motivational barriers (e.g., being too lazy to cook after a busy day at university). In addition, knowledge/information-related barriers, social-support-related barriers, and transition-related barriers emerged from our interviews. The variety of barriers addressed and the different views on some of these, indicate that various strategies seem to be needed to improve the eating behavior among university students and to prevent them from gaining weight and developing non-communicable diseases in the future.

## 1. Introduction

It is widely acknowledged that healthy eating can prevent people across all age groups from weight gain and developing overweight/obesity [1,2]. Furthermore, following a healthy diet can be helpful in preventing the occurrence of various non-communicable diseases such as type 2 diabetes and cardiovascular disease [1]. However, unhealthy eating habits have been commonly found among university students in different countries worldwide [3]. For example, high fast food consumption, low consumption of fruits/vegetables, and irregular meal patterns such as skipping breakfast and frequent snacking have been reported [4,5,6,7,8,9]. Few studies have examined the eating habits of university students from Germany, but existing studies suggest that especially the low consumption of fruits and vegetables (between 70%–95% of students do not meet the recommendation of eating five or more portions a day) is of concern [4,10,11]. In addition, two of these studies report that particularly male students consume fast food several times per week [4,10]. In line with other Western societies, higher education is a main prerequisite to obtain well-paid job opportunities [12]. Currently more than half (57%) of all young adults that finish secondary education in Germany begin university studies [13]. Due to the increasing numbers of university students in Germany, it is important to examine health behaviors of this population in order to improve their health outcomes [14]. The transition from school to university has been found to correspond with changes in various health behaviors including healthy eating [15,16,17]. Changes in eating behaviors during the transition from secondary school to university were reported by approximately 65% of all students in a sample of students (n = 689) from more than 40 universities across Germany. Additionally, more than half (55.3%) of these students reported a decrease in regular meals after the transition from secondary school to university [4].

In the US, living on the university campus with easy access to “junk foods” and exposure to “all you can eat” dining halls have been reported as reasons for changes in eating behavior [18,19]. However, university students in Europe usually do not live on campus [14,18,20]. In contrast, students in Germany and other European countries such as Belgium or the Netherlands live either independently, together with other students in student residencies, or with their parents [14,18,20]. Findings from previous European studies revealed that eating behavior differs between students that still live with their parents and those who have left hometown for studying: Students who still lived with their parents consumed healthier diets [3,21]. After leaving their parents’ home to start studying, most students are responsible for planning, preparing and cooking their meals on their own in European countries [21,22]. Although students can visit the university canteen to eat lunch—in our previously published study more than half of all students (55.2%) report regularly eating lunch at the university canteen [4]—there are no “all you can eat” buffets like in the US. Furthermore, in various European countries (e.g., Germany, France) the education system of universities is quite different from the school system and less structured than in the US. In addition, in Germany students are responsible for organizing their whole university day including arranging their class schedules on their own, which might also influence their eating behaviors (e.g., due to a lack of time).

Moreover, studies indicate that unhealthy eating behaviors established during the university period seem to persist into later life, and thus may result in poor long-term health outcomes [3,9]. Therefore, strategies to improve the eating behavior in this subgroup of young adults seem necessary. However, to ensure the success of such strategies, it is important that they are tailored to the needs of the target population [23,24,25]. Here, a qualitative approach is the method of choice, as it provides a deeper insight into the views, attitudes, and needs of the target population. In the past, qualitative methods have been used as a groundwork for the development of health promotion strategies [24,26]. A deeper understanding of the barriers that hinder university students from following healthy diet is necessary and may be particularly helpful to develop effective strategies that improve the eating behavior among university students [1,27,28].

Former research on barriers to healthy eating among university students could identify various barriers including: Lack of time [4,27,29,30,31,32,33,34,35], motivation [24,27,35], social support [27,29,31,35,36,37], and financial barriers [4,27,29,30,31]. However, most studies focused on barriers in freshmen students and only little is known on barriers affecting students in higher semesters [27]. Findings from our previously published quantitative study indicate that students in higher semesters seem to be affected by different barriers than younger ones, probably because the older students have learned to cope with various barriers (e.g., missing skills to cook a healthy meal) over time [4]. Therefore, views of students from higher semesters on barriers to healthy eating might enrich the opinions of younger students [27] that were found in previous studies. Moreover, most studies on barriers to healthy eating have been conducted in the USA and Australia [28], but little research has been done in European countries [27,35,38] including Germany [4]. Due the above-mentioned lifestyle and cultural differences that exist between continents and its university contexts, findings on barriers to healthy eating cannot be transferred from one continent to another [16,18,27,39].

Therefore, studies that focus on barriers affecting the eating behavior of university students in European countries are needed. We thus conducted a qualitative study to get a deeper understanding on the barriers that hinder German university students in particular from following a healthy diet.

## 2. Materials and Methods

The manuscript reports on qualitative data from our Nutrition and Physical Activity in Adolescence (NuPhA) Study. The NuPhA-Study has a mixed-methods research design and consists of a quantitative online-survey including university students from more than 40 universities across Germany (*n* = 689) followed by semi-structured qualitative interviews (*n* = 20). Quantitative results on barriers to healthy eating have already been published [4].

The qualitative study phase was conducted between 03/2016 and 12/2016. It included a convenience sample of 20 university students that were enrolled at universities from the Rhine-Neckar region, located in the South-West of Germany. Students were recruited by distributing flyers and sharing the call for participation via social networks. Inclusion criteria for the qualitative study phase were: (1) being enrolled at a university for at least one semester and (2) no longer living with one’s parents. After conducting 20 interviews, we felt that theme saturation had been reached and therefore recruitment was ended. All interviews were conducted face to face by the first author, who is familiar with conducting qualitative interviews, with no third person present in the room. We used a semi-structured interview guide that included questions on: Barriers to healthy eating, the role of the university context for healthy eating, and ideas that could help them to make healthier food choices in the future during the university day (Table S1). All students gave their written informed consent before participating. Prior to the start of the interviews, students filled out a brief questionnaire including demographics (e.g., age, sex), details on their studies (e.g., study course, study degree), and information on eating behavior (e.g., if they try to follow a healthy diet). Every student received a gift card worth 20 € as an incentive. The NuPhA-Study was approved by the Ethics Committee of the Medical Faculty Mannheim, Heidelberg University, Germany (2013-634-N-MA).

All interviews were audio recorded (Olympus-DS-2500) with the students’ consent and transcribed verbatim. Interviews ranged between 28 and 59 min (mean: 41:45 min). Qualitative content analysis according to Mayring [40] was applied to identify themes, consistencies, and contradictions across all interviews. We developed an initial set of main codes based on the interview guide. These main codes were further refined during the coding process by adding codes and subcodes that emerged from the interview material. All interviews were independently coded by two researchers (J.H.-K., H.D.) with the assistance of MAXQDA 12.3 software for Windows (VERBI Software GmbH, Berlin, Germany). The agreement between coders was high (82%).

## 3. Results

### 3.1. Sample Characteristics

University students that participated in the qualitative study were between 20 and 26 years old (mean age: 22.8) and 65% were female (*n* = 13). Most students (*n* = 14) studied social sciences. Nine students were undergraduate students between their third and sixth semester (Table 1). While most students (*n* = 18) reported that they try to follow a healthy diet, only six students stated that they find it easy to follow a healthy diet.

### 3.2. Barriers to Healthy Eating

Our qualitative analysis resulted in the following six barrier categories: Time-related barriers; Environmental barriers; Social inclusion/social support-related barriers; Motivational/attitudinal barriers; Knowledge/information-related barriers; Transition-related barriers. Further details on the students’ views for each of the barrier categories identified is provided in the following paragraphs.

#### 3.2.1. Time-Related Barriers

A main barrier to healthy eating that emerged from our qualitative interviews was lack of time due to university commitment (S01, S02, S04, S05, S06, S07, S08, S09, S13, S17, S15, S18, and S20). Some of those students stated that especially planning (e.g., deciding what to cook and food shopping) and preparing a healthy meal was *“really time consuming”* (S15, S04, S13, and S18). Students also felt that university life was so stressful that they hardly found time to eat (S09, S01, S04, S06, and S15) with one student mentioning that all she could think of was: *“Oh god, I have to eat something, but where can I get something quickly?”* (S09). However, some different views also emerged during our interviews with other students mentioning that *“lack of time is not playing such a big role”* to them (S16, S03, S10, S14, and S19), with even one student stating that *“I think cooking is relaxing and I take the time to do it”* (S14). In addition, two other students expressed skepticism about time-related barriers and emphasized these as *“an excuse* [for not following a healthy diet] *in most cases”* (S11, and S12).

#### 3.2.2. Environmental Barriers

Several environmental barriers to healthy eating were also addressed. Eating at the university canteen was named by many students as a barrier to healthy eating (S01, S02, S04, S05, S06, S08, S09, S10, S13, S15, S19, and S20). For example, one student stated: *“Well, when I’ve spent the whole day at university, then I have probably not been eating very healthy that day, because I’ve eaten at the university canteen, and personally I think the meals there are not quite healthy”* (S20). Although several students noted that there are healthy meal options at the university canteen, they often mentioned the bad taste or the low quality of these meals (S01, S02, S05, S10, and S20). Moreover, students also felt unsure about the ingredients and assumed that many meals contain a lot of flavor enhancers (S02, S04, S05, S09, and S19). In addition, many students perceived that healthy meal options were offered at higher prices than the typically offered standard meals (S06, S08, S10, S13, S14, S15, and S17). One student stated: *“When you go to the canteen it doesn’t mean that you will eat a healthy meal, because you always eat schnitzel and fries. And if you want to eat something healthy, the meal will be much more expensive, and costs more than I am willing to pay”* (S06). Other students mentioned that the opening hours of the university canteen did not fit their university schedules (S01, S19) and some also missed healthy snacks such as fruit which they could take away and eat in the short breaks between their classes (S01, S02, S09, S13, and S17).

Besides the higher costs of healthy meals at the university canteen, views about financial reasons as a general barrier to healthy eating differed. While some students named the higher costs of healthy food as a barrier (S10, S17, S15, and S20), several students emphasized that financial reasons were not a barrier to them (S02, S04, S05, S07, S08, S13, S14, S16, S18, and S19). Although some of these students mentioned that they did not regularly shop organic food products due to their high prices, they felt that healthy meals like fruit and vegetables were also available at a discounter store: *“At* [name of the food store]*, that’s an organic food store, everything is very expensive and I think I could not afford to buy all my groceries there. But in general, I think, you can eat healthy without having to spend much, you can also buy vegetables at* [name of a German discounter] *or something”* (S05). Students who had spent a semester abroad (S14, S07) particularly emphasized the low prices of healthy foods in Germany compared to other countries such as Norway or the USA: *“In the USA it’s* [healthy food] *really damn expensive, there a pepper costs $2.50 and then you think twice, whether spending your money on it or not. […]. But here [in Germany] financially, it’s not a barrier at all, I even think it’s cheaper to cook fresh meals at home instead of going to the university canteen”* (S14). Furthermore, the various opportunities to buy healthy food in Germany were mentioned (S03, S12, S18, and S19). For example, one student felt that *“in Germany* [compared to the USA] *it’s really easy to follow a healthy diet, there are fresh products, there are farmers markets where you can go […] so there is no barrier to me”* (S12). However, short shelf life of healthy food turned out to be problematic for some students, stating that *“When I buy fresh products, I always need to pay attention that it doesn’t go to waste”* (S16, S01, S06, S15) and *“often you can’t buy vegetables or meat in portions for a single person […]. So that’s one reason for me to say ‘Okay, then I’ll simply eat a sandwich’, instead of cooking something that I then would have to eat for the next three days”* (S01).

#### 3.2.3. Social Inclusion and Social Support-Related Barriers

Students felt that *“somehow it’s not worth cooking for just one person”* (S15, S01, and S02) and that they *“have to eat healthy when they are together with others”* (S02) suggesting that cooking and eating alone were also barriers to healthy eating. In contrast, other students named social inclusion as a barrier to healthy eating (S03, S05, S07, S18, and S20). For example, students mentioned going to university canteen to *“be among people and talk to [their] friends”* (S05) and *“going out to eat with friends, for example, a pizza, instead of cooking a healthy meal at home alone”* (S20). However, several students emphasized that support from friends and family was helpful for them to follow a healthy diet (S02, S04, S06, S08, and S15). One student stated: “*What really helps me* [to follow a healthy diet] *is my environment, my friends which pay a lot of attention to what they eat. I cook a lot with my best friend, she has a really big influence on me”* (S15).

#### 3.2.4. Motivational and Attitudinal Barriers

Motivational/attitudinal barriers were also addressed during our interviews with several students reporting difficulties in *“overcoming the inner temptation”* (S07, S10, S15) and *“a lack of resilience”* (S19, S04). In particular, students reported to *“be a sucker for sweets”* (S15) and viewed sweets and chocolate also as *“something that you need from time to time when you realize, it* [studying] *isn’t running so smoothly anymore”* (S10) and *“to motivate* [yourself] *and to get energy again”* (S07). Other students mentioned *“being too lazy”* (S06) as one barrier to healthy eating and found it *“easier just to get something [to eat] along the way instead of cooking”* (S17) or stated that *“after finishing all their tasks for university, [being] already hungry and no longer motivated to […] go grocery shopping or to cook something”* (S04). This indicates that motivational barriers might be related to time-related barriers. The taste of healthy food was also addressed with students declaring themselves to be *“not such a big fan of vegetables”* (S17), and *“the recipes, dishes, that are generally considered to be healthy, I just don’t like the taste of it”* (S05) whilst *“there are so many unhealthy things that are just delicious such as pizza”* (S07). Another motivational barrier that emerged during some interviews was a lack of awareness of the importance of a healthy diet (S04, S11, S12, S17). For example, one student emphasized that *“if you don’t have severe weight problems you do not pay so much attention to it* [healthy eating]*, even though you should. I mean, I don’t want to get diabetes or something like that later”* (S12).

#### 3.2.5. Knowledge and Information-Related Barriers

In addition, knowledge and information related barriers were identified in our qualitative interviews. For example, several students (S01, S04, S09, S11, and S19) stated to have a lack of knowledge about healthy eating. Students mentioned that it is difficult to evaluate their dietary behavior due to *“not having a particularly in depth knowledge on nutrition”* (S19) or *“knowing too little about it* [the meaning of a healthy diet]*”* (S11). One student realized just during the interview that *“I do not know what is healthy, and what I have to do to be able to say ‘I’m following a healthy diet’.”* (S01). Other students mentioned a *“lack of information”* (S11), or the need to *“inform* [oneself] *a bit more about what contributes to a healthy diet”* (S16). In addition, another student emphasized that *“nutritional information on food packages are often difficult to read and you have no motivation or time to study all these in order to find out how much sugar or other things it contains”* (S07). A lack of cooking skills also emerged in some interviews with students mentioning that they *“do not trust in* [their] *own cooking skills”* (S04) resulting in a missing variety of meals *“because I cannot cook as good as my mom”* (S19). However, one student who was already in a higher semester felt that cooking skills *“have improved a lot* [since starting studies] *due to the increasing experience”* (S09), indicating that a lack of skills may be especially an issue when starting studies.

#### 3.2.6. Transition-Related Barriers

Some additional transition-related barriers got obvious from our interviews. For example, a missing routine in everyday life got obvious with one student mentioning: *“Every week, every day being more or less different over and over again, and having no structured procedures, that what is making it really difficult for me”* (S06). Other students were also struggling with lecture hours every day of the week, which prevented them from eating regular meals: *“Always eating at different times that´s something [they] do not come along with”* (S05, S04, S01). One student emphasized in contrast to extended and relaxed family meals, when eating alone at the student’s flat was *“something that is usually done while doing something else. For example, I read something and eat while doing that […]. It [eating] is something that needs to be done but not really something that you can enjoy”* (S01), indicating that the value of eating has changed since the transition to university.

### 3.3. Possible Strategies to Overcome Barriers to Healthy Eating

Students were asked for ideas that could help them to make healthier food choices during the university day in the future. The following two main strategies emerged from our interviews: (1) Improving the on-campus food environment; (2) Promotion of healthy eating within the university setting.

#### 3.3.1. Improving the On-Campus Food Environment

Most ideas centered on improving the university canteen. Students suggested to offer healthier meals at lower prices to make healthy options like salads more attractive for students (S06, S07, S08, S10, S13, S15, and S17). In addition, students emphasized that more fresh, tasty, and high-quality meals should be offered (S01, S02, S06, S08, S10, S13, S15, and S20). Suggestions included reducing salt, flavor enhancers, and offering more fresh and appealing vegetables. Furthermore, a broader variety of healthy meals and especially offering more vegetarian/vegan meals was suggested (S04, S06, S07, S08, S09, S13, and S20). Students also mentioned that offering healthy meals at a buffet, where they could arrange their meals on their own would be helpful (S07, S09, S10, and S13). For example, one student emphasized: *“I would say, it would be quite cool, if we had a buffet, where you can choose the things you eat yourself. But really offer a lot of tasty and healthy things and not only overcooked broccoli, because then nobody eats that, then you prefer to eat the schnitzel”* (S13). Students also felt that replacing unhealthy snacks such as chocolate bars or ice cream with healthy snacks such as apples or fruit smoothies might improve on-campus eating behavior among university students (S01, S02, S13, and S17).

#### 3.3.2. Promotion of Healthy Eating within the University Setting

Besides improving the offer of healthy meals at the university canteen students also suggested that universities should provide information on healthy eating, for example by offering online courses on different nutrition topics (S04, S11, S12, and S19): *“They [the universities] could offer nutrition courses, just like they offer language courses, only three to four seminars”* (S19). Another student perceived that the university could start health promotion campaigns to improve the eating behavior of students: *“They [the universities] could promote it [healthy eating] a bit more proactively, they could say ‘Please care about your eating behavior’*. *They could see it as active marketing or also as a duty of the university to say, ‘Look what we are offering [at the university canteen], and what you can do to improve healthy eating’”* (S11). In contrast, other students perceived that the university was not the right place to target the eating behavior of students and felt that everybody would be responsible for following a healthy diet (S06, S18): *“The university has nothing to do with nutrition or with a healthy diet, I mean, if you want to eat something healthy, you can do that at home or just bring your healthy meal with you”.*

## 4. Discussion

### 4.1. Main Findings and Comparison with Previous Research

Our qualitative findings revealed that students are especially affected by a lack of time due to university commitments and by environmental barriers (e.g., a lack of cheap, tasty, and healthy meal options at the university canteen). In addition, knowledge/information-related barriers, social-support-related barriers, and transition-related barriers were observed in our interviews.

Time-related barriers were also reported in former studies conducted in various countries [4,27,29,30,31,32,33,34,35]. In line with other studies [29,30,35,37] it became obvious from our interviews that shopping, preparing, and cooking healthy meals were perceived by students to be time consuming and therefore difficult to incorporate into a busy university schedule. Moreover, our findings indicate that time constraints due to busy university life seem to affect several motivational/attitudinal barriers. For example, after an exhausting university day, students were no longer motivated to go shopping or to spend time on cooking a healthy meal. Thus, besides time management courses [34], strategies that enable students to prepare healthy meals with less efforts seem necessary [28]. Some useful strategies to overcome this barrier could be to install on-campus shopping locations or providing students with recipes for quickly and healthy meals.

Another important barrier was a lack of healthy meals at the university canteen. As time constrains were reported in our previous published quantitative study to be a main reason for eating at the university canteen [4], improving the availability of healthy meals at on campus eating locations seems to be another potential starting point to improve student’s eating behavior. In line with findings reported by former qualitative studies [27,37], students in our qualitative sample emphasized that besides a higher availability of healthy snacks, especially the taste and the quality of already available healthy meals need to be improved to make healthy meals more attractive to students. In line with findings reported by Deliens et al. [27], our qualitative data made it obvious that offering healthy meals at lower prices would be an important requirement to achieving healthier food choices at on-campus eating locations. First evidence from intervention studies showed that pricing strategies at university canteens such as higher prices for fast-food and lower prices for fruits were effective in improving the eating behavior among students [41,42]. In previous research, high costs of healthy food were identified as another important barrier [4,27,29,30,31]. However, views in our qualitative study on this topic differed. While some students perceived that unhealthy food were offered at lower prices than healthy food, others emphasized that everyone can afford to buy healthy food in Germany. Further research might be necessary to explain these varied findings and to effectively address financial barriers among students.

Different views were also found for social aspects, which is in line with former research [27,29,31,35,36,37]. While some students felt that going out for lunch or dinner with friends was a barrier for them, others emphasized that support from partners and friends enabled them to follow a healthy diet. Ashton et al. [29] suggested that positive social aspects such as social support and social inclusion should be considered when planning future interventions on healthy eating for young adults. Some studies in adolescents and young adults identified positive associations between friends’ support on weight-related health behaviors such as healthy eating [43,44,45]. As some students in our qualitative study felt that it is not worth the effort to cook only for themselves, strategies that focus on social inclusion might also be helpful to overcome this barrier in the future.

In our former quantitative manuscript, we found that a lack of knowledge or information seemed to only affect a minority of students [4]. However, when students started thinking about the meaning of healthy eating for an extensive amount of time during our qualitative interviews, some realized that they do not exactly know what is meant to eat a healthy diet and that they would need more information on this topic. This might indicate that students tend to overestimate their knowledge on nutrition in quantitative questionnaires. For example, Matthews et al. [46] found that while students reported to care about healthy eating, they had difficulties in meeting dietary guidelines and often were not able to correctly report such guidelines. Some students in our qualitative sample suggested nutrition education classes might be useful to overcome knowledge/information-related barriers which was also suggested by students in the study of Deliens et al. [27]. In addition, starting healthy eating campaigns at on campus eating locations may also be effective in improving the eating behavior among students [47]. Another barrier identified in our interviews was a lack of awareness of the importance of a healthy diet. This tendency was also identified by a study from Scotland but in line with our findings, even concerns regarding one’s own health did not enforce healthy eating [35]. In addition, former research revealed that most students do not even recognize that their current health behaviors may affect their future health status [46,48] and thus interventions addressing future health turned out to be ineffective [49]. Therefore, negative consequences of an unhealthy diet which affect students’ current health (e.g., weight gain) should be addressed in nutrition classes, instead of future health [28,46].

Transition-related barriers also emerged from our interviews. For example, students reported to be struggling with missing routines in everyday life since transition to university resulting in negative eating behaviors. Such transition-related barriers were also reported in two former qualitative studies [27,36]. Clusky et al. [36] report that students did not expect that starting university would affect their previously established lifestyle behaviors. Therefore, strategies such as mentoring programs or peer support that better prepare students for the changes that go along with the transition from school to university seem to be necessary to enable students to maintain or establish healthy eating behavior throughout university [14].

### 4.2. Limitations

Our study was not without limitations which should be considered while interpreting our study’s findings. As our qualitative study consisted of a convenience sample recruited in a single region of South-West Germany, generalizability of the findings may be limited. For example, views about barriers to healthy eating and ideas suggested to improve their eating behavior, might differ when asking students from other regions in Germany. However, our study provides valuable insights on barriers affecting the eating behavior of university students, which can serve as a potential starting point for promoting healthy eating among university students in the future.

## 5. Conclusions

Our qualitative findings suggest that students were especially affected by time-related barriers and environmental barriers. In addition, knowledge/information-related barriers, social-support-related barriers, and transition-related barriers emerged from our interviews. The variety of barriers addressed and the different views on some of these, indicate that various strategies seem to be needed to improve the eating behavior among university students and to prevent them from developing obesity and non-communicable diseases.

## Figures and Tables

**Table 1:** Characteristics of students participating in the qualitative part of the Nutrition and Physical Activity in Adolescence (NuPhA) -Study (*n* = 20).

Characteristics	*n*
*Age (years)*	
20–23	16
24–26	4
*Sex*	
male	7
female	13
*Study course*	
social sciences	14
medicine	3
law	2
teaching	1
*Study degree (semester)*	
Bachelor’s degree (semester 3–6)	9
Master’s degree (semesters 2–6)	6
state exam (semesters 7–10)	5

## Supplementary Materials

The following are available online at https://www.mdpi.com/2072-6643/11/10/2440/s1, Table S1: Interview guide applied during the qualitative part of the NuPhA-Study to conduct the face-to-face interviews.
