# Supplementary material for: ‘Oh God, I Have to Eat Something, But Where Can I Get Something Quickly?’—A Qualitative Interview Study on Barriers to Healthy Eating among University Students in Germany
Source: Nutrients. 2019 Oct 14;11(10):2440. doi: 10.3390/nu11102440 (PMC6835904; doi:10.3390/nu11102440)
Supplement: Supplementary file 1 [file nutrients-11-02440-s001.pdf]

Table S1: Interview guide applied during the qualitative part of the NuPhA-Study to conduct the face-to-face interviews

Opening question

- **At first, I want to get to know a little bit more on your current diet. Please tell me how your daily diet typically looks like?**
  - How often do you consume fruit and vegetables?
  - How important is it for you to have breakfast in the morning?
  - How important is it for you to consume regular meals?

Questions on the meaning of a healthy diet

- **What do you personally mean by a healthy diet?**
  - What role does a healthy diet play for you?
  - To what extent would you say you follow a healthy diet?
  - Is it easy for you to follow a healthy diet? In what way?

Question on barriers to healthy eating

- **For you personally, what would you say are the most important barriers to healthy eating?**
  - What would you say is the main barrier to you?
  - What about time-related barriers? And especially, a lack of time due to university commitment, how does this barrier affect your eating behavior?
  - What would you say, how important are financial reasons as a barrier to healthy eating for you personally?
  - Are their any other barriers that prevent you from eating a healthy diet? If yes, please tell me more about these additional barriers?

Questions on strategies to overcome barriers in future

- **When you think of the general conditions at your university, to what extent do they support you in following a healthy diet?**
  - Do you regularly eat at the university canteen?
  - Are healthy meals offered at the university canteen?
- **Do you have an idea who or what could help you improve your diet in the future?**
  - Do you have ideas how the university could support you in following a healthy diet?
  - Do you have ideas how the university canteen could support you in following a healthy diet?
  - What other ideas come to you mind that could help you in following a healthy diet?

Closing question

- **Is their anything else that you want to add to the themes that we talked about?**

Comment: As all interviews in the NuPhA-Study were conducted in German language the original interview guide also was written in German. For the interview guide presented in this supplement the original interview guide was translated into English.
